# Has the COVID 19 Virus Changed Adherence to Hand Washing among Healthcare Workers?

**DOI:** 10.3390/bs11040053

**Published:** 2021-04-15

**Authors:** Rosalia Ragusa, Marina Marranzano, Alessandro Lombardo, Rosalba Quattrocchi, Maria Alessandra Bellia, Lorenzo Lupo

**Affiliations:** 1 Health Technology Assessment Committee, University Hospital “G. Rodolico”, Via Santa Sofia, 78 95123 Catania, Italy; 2 Department of Medical and Surgical Sciences and Advanced Technologies “G.F. Ingrassia”, Via Santa Sofia, 87 95123 Catania, Italy; marranz@unict.it (M.M.); l.lupo@policlinico.unict.it (L.L.); 3 Committee for the Control of Hospital Infections, University Hospital “G. Rodolico”, Via Santa Sofia, 78 95128 Catania, Italy; lombardo.alessandro@policlinico.unict.it; 4 U.O. Health Education, University Hospital “G. Rodolico”, Via Santa Sofia, 78 95128 Catania, Italy; quattrocchi@policlinico.unict.it; 5 School of Specialization in Microbiology, University of Catania, Via Santa Sofia, 78 95123 Catania, Italy; alessandra.bellia03@gmail.com

**Keywords:** hand hygiene, adherence to hand washing, health promotion positive actions, hand-washing permanent monitoring, COVID 19, HCWs behavior, guideline adherence

## Abstract

The aim of the study was to assess adherence to hand washing by healthcare workers (HCWs) and its variations over time in hospital wards. We wanted to check whether the pandemic had changed the behavior of HCWs. The study was conducted between 1 January 2015, and 31 December 2020. The HCWs were observed to assess their compliance with the Five Moments for Hand Hygiene. We described the percentage of adherence to World Health Organization (WHO) guidelines stratified per year, per specialty areas, per different types of HCWs. We also observed the use of gloves. Descriptive data were reported as frequencies and percentages. We observed 13,494 hand hygiene opportunities. The majority of observations concerned nurses who were confirmed as the category most frequently involved with patients. Hospital’s global adherence to WHO guidelines did not change in the last six years. During the pandemic, the rate of adherence to the procedure increased significantly only in Intensive Care Unit (ICU). In 2020, the use of gloves increased in pre-patient contact. The hand-washing permanent monitoring confirmed that it is very difficult to obtain the respect of correct hand hygiene in all opportunities, despite the ongoing pandemic and the fear of contagion.

## 1. Introduction

Hands are the main vehicle of infection in healthcare. The increasingly frequent use of digital devices multiplied the opportunities for contact between hands and the environment. The repeated pressures of the fingertips, sometimes covered with gloves and sometimes not, on cell phone or on workstation keyboards, PCs, monitors, or buttons of electro-medical equipment makes these objects become vehicles of infections [1,2] Recent evidence showed the possibility of coronavirus transmission after contact with a contaminated dry surface [3].

Even though the World Health Organization (WHO) and Centers for Disease control the guidelines and recommendations for appropriate hand hygiene (HH) practices since 2002 [4,5], the compliance with this practice remains poor, in the majority of cases [6].

Numerous studies tested the knowledge, attitudes, and HH compliance among healthcare workers (HCWs), through questionnaires or by direct observation of the HCWs behavior [7,8,9,10]. It was reported that the growing awareness of HCWs on the importance of hand washing causes a reduction of about 30% in the transmission of infectious agents [11].

Despite extensive data that were published on hand washing and on the usefulness of this procedure in preventing the transmission of infectious agents within the hospital, the topic is not discussed as such to be an indicator of the behavior of health workers towards patient. Some authors evaluated the evidence on behavior change interventions and policies directed at healthcare professionals working in primary healthcare centers. Education, training, and enablement in the context of collaborative team-based approaches are effective for changing the practice of primary health care professionals [12,13]. The characteristics of successful behavior change interventions in healthcare are unclear [14].

The interventions are commonly divided into three main categories—persuasive; action and monitoring; and educational or informative [15]. Audit and feedback are widely used as a quality improvement tool, based on the belief that professionals are prompted to modify their practice when performance feedback is given [16]. We consider that this model, characterized by objective measurements of professional practice in a healthcare setting, is the most useful intervention to change health professional behavior and hand washing observations are part of a planning prompts to change behaviors, in our hospital.

In this work, we described what was observed over a period of 6 years and verified the different approaches to the same hand-washing hospital procedure. In particular, we wanted to verify if the percentage of adherence to hand washing was influenced by the COVID 19 pandemic, in 2020.

Recent studies published in the literature report that the vulnerability to mental health illnesses increased in the general population [17,18,19,20,21] and in frontline healthcare professionals [22,23,24,25], during the COVID-19 pandemic.

We reported observations of a routine practice among health professionals to verify whether the fear of acquiring the infection, nervousness, anxiety, social distancing, or other psychological aspects, unknowingly led to the adoption of proper behavior, consequently improving the level of adherence to the hand washing procedure.

Assuming that external elements can affect healthcare workers’ adherence to hand washing, their presence or absence was recorded, during the observations in the wards. We checked whether the compliance of the procedure was linked to scientific knowledge or whether other factors acted in increasing or reducing the percentage of adherence to hand hygiene, in addition to permanent monitoring of hand washing.

## 2. Materials and Methods

This study was conducted between 1 January 2015 and 31 December 2020, in a reference teaching hospital of 400 beds located in Catania, Sicily, Italy. Observation of the HCWs for the hand washing practice was conducted during one of their contacts with the patients in the different wards, by members of the infection control team. The HCWs were observed during working shifts to assess their compliance with “Five Moments for Hand Hygiene” extracted from the WHO Guidelines on Hand Hygiene in Healthcare [4]—before touching a patient or before having contact with an object belonging to the healthcare area; before a clean/aseptic procedure; after body fluid exposure; after touching a patient, and after touching patient surroundings.

The observers were infection-control nurses who had done a training course according to the rules of the WHO. They were inside the room looking at the behavior of HCWs, while they were taking care of the patients. WHO form checklist was used to collect data obtained by direct visual observation on compliance with HH guidelines and proper glove or gown use, on at least 200 opportunities per ward, every six months.

For this study, we grouped members of the healthcare team that provided direct medical care to patients, according to their main mission, and labelled them as follows.

NU–Professional nurses. The people in this group provide direct, hands-on patient care, often directed by a doctor, but also initiating care based on their own clinical judgment and observation at the patient’s bedside. They monitor patient progress and response to treatment. They deliver primary, clinical, bedside care to patients in hospitals, including nursing assessments, medication administration, and delivery of blood products. They provide care for all types of patients, supervised by a head nurse.

PHY-Attending Physician or Specialty doctors. They are medical doctors who graduated from an accredited medical school, with extra expertise in one type of medicine or another. Attending Physicians are doctors who are responsible for supervising and teaching, and training interns, residents, and medical students. They are ultimately responsible for all aspects of patient care.

R-Resident Physician or Medical Student. Resident Physician is a physician who participates in a postgraduate medical education and training program in a specialized area of medicine. He acts as both a student and a health care provider and works in concert with other members of the health care team to provide direct medical care to patients. He is supervised by attending physicians or Specialty doctors.

Others-Support Staff. In this category, we grouped healthcare assistant or auxiliary nurses. They have a lower level of training than a professional nurse, typically providing basic patient care. Auxiliary nurses require no academic qualifications. The role of an auxiliary nurse is to assist qualified nursing practitioners in administering care to patients. They are required to assist patients for personal care, for food, and personal hygiene.

Each year the observations were made in a limited number of wards, which were randomly selected at the beginning of the year. The list of potential wards always included the departments of general medicine, surgery, and intensive care. Surveys of other departments, such as pediatric departments, were carried out annually but did not form a part of this work.

The personnel knew that they were observed, but they did not know exactly when. Everyone was made aware of the results of the observations carried out. A letter from the Hospital’s Infection Control Team was addressed to the Medical Director and the Head Nurse at the beginning of the year, informing them that they would be part of the control program.

The following interventions were carried out to increase adherence to HH, as a part of the Hand Washing Observation audit and feedback program (HWOP):

- HCWs training and field training, in small groups, were carried out in various departments with simulators.
- Observations were carried out in wards for two months, twice a year, and the results were reported to the Medical Director and Head Nurse to share the recorded data with their staff
- Fixed hydro alcoholic gel dispensers were installed in the main transit points of various departments.
- Posters were affixed to remember how to correctly perform hand hygiene, as ordered by the WHO.
- Informative brochures with the correct instructions for hand hygiene were edited for staff and visitors, annually renewed, and distributed on the celebration day on 5 May.

The study consisted of two phases—in the first phase, the observations made in a single unit were reported and then grouped into four clinical areas.

We also recorded, once for each session of observation, the presence or absence of favoring factors concerning adherence to HH in a special note field. We assigned one point when the factor was present and zero when the factor was absent.

### 2.1. Study Endpoints

The primary endpoint is the overall percentage of adherence to WHO guidelines and recommendations for appropriate HH practices over a six-year period by HCWs. We described the percentage of adherence to WHO guidelines stratified per year, per specialty area, and for different types of HCWs. We wanted to check whether the use of gloves in pre-contact maneuvers with the patient showed changes, by comparing the data observed in 2019 and those observed in 2020, because of COVID 19.

### 2.2. Statistical Analysis

Descriptive data were reported as frequencies and percentages. One-way analysis of variance along with orthogonal *t* test comparisons were performed among group means.

*p* values of less than 0.05 were accepted as significant. Trend analysis along years using linear correlation was conducted among operators working in different setting units.

## 3. Results

We observed 13,494 hand hygiene opportunities in total.

### 3.1. Hospital’s Global Compliance

The hospital’s global hand hygiene compliance per year is described in Table 1.

The percentage of overall hospital compliance with hand washing increased sharply in 2016, when the systematic intervention actions described in the methods began. The percentage grew further in recent years, increasing overall by 20% in 6 years. However, it is not possible to evaluate the weight of each single intervention in a reasonable manner.

Hand washing with soap and water is preferred to hand rubbing with an alcohol-based formula. Alcohol-based hand rubs rather than soap and water are preferred by physicians and residents.

The hospital’s global percentage of adherence to the procedure did not change in 2020.

In 2019, 1652 pre-contact opportunities were observed and in these, the use of gloves was recorded in 68% of cases. In 2020, with 1188 pre-contact opportunities, gloves were used in 74% cases. The overall percentage of use of gloves, during patient care, increased slightly. It was noted that in 2020, the use of gloves, previously reserved only for aseptic maneuvers, increased in pre-patient contact, going from 66 (2019) to 73 (2020). On the contrary, during aseptic maneuvers, the use of gloves reduced (from 89% to 82%), while the use of a gel hydro-alcohol increased.

### 3.2. Adherence by Year and by Professional Category

Adherence to HH by year and by professional category is described in Table 2.

The majority of observations concern nurses who are confirmed as the category most frequently involved with patients. Nurses recorded higher adherence (*p* < 0.05) to the procedure, except in 2018. The percentage of adherence remained stable throughout the years under observation. 

During the pandemic period, we observed a reduction in observations among residents who were not allowed on the wards. The lowest adherence was registered, every year, between social healthcare assistant and nursing support personnel.

The percentage of adherence of residents is generally lower than that of doctors’, while the percentages of adherence of the social healthcare assistants were always lower than those of nurses. Surgeons are more sensitive to the problem than internists and anesthetists.

### 3.3. Adherence by Areas

Hand washing adherence was differentiated by the type of departments; shown in Table 3.

No significant differences were found between the different areas.

Adherence to HH per area, professional category, and year is detailed in Table 4.

The surgical activity increased to accommodate non-COVID patients from other hospitals.

**Medicine**—The percentage of global adherence of nurses showed a slow but steady linear growth showing strong correlation with time, with an R^2^ of 0.97. The Hematology Unit, opened in 2018, increased the global average of adherence up to 50% of the corrected actions. As a matter of fact, the Hematology Unit compliance was 85%, showing its great attention to the procedure.

**Surgery**—The percentage of adherence to the hygiene of the hands of nurses sometimes exceeded that of the medical personnel (surgeons). Compliance showed variations over the years in all professional categories observed.

**Intensive care**—Nurses in the ICU always showed higher compliance, in comparison with the other professional figures observed. Anesthetists sometimes reported insufficient rates.

### 3.4. Environmental and Organizational Factors

The environmental and organizational factors, mostly influencing the aspects of the procedure are indicated in Table 5.

As we can see in Table 5, in all departments, medical directors and head nurses always received the reports of observations on HH carried out on their operators. Each final report included comparative data with previous ones as an effective way to change behavior, such as efforts to accomplish best practices.

The hospital annually offers several editions of a training course with videos, questionnaires, and tests, with a simulator. New workers or those serving in high-care-intensive wards are especially welcome to participate. In 2019, the members of the Hospital Infection Control Committee changed and it was not possible to organize these courses, consequently, these were postponed until the following year. Unfortunately, due to the pandemic in 2020, the hospital was able to organize only a few courses, in the first 2 months only.

The medicine area showed a steady increase over the years, starting from 3 up to 8 positive factors listed, until 2019.

A correlation was sought between the score obtained by the areas and HH compliance for the different years observed—medical area *r* = 0.6; surgical area *r* = 0.3; intensive care area *r* = 0.23. For the relatively small number of observations only in the medical area, a weak positive correlation was found, even if it did not reach a significant level. No correlation was observed in the other data sets.

## 4. Discussion

The recent COVID-19 pandemic focused on proper HH practices (social washing, disinfection with a hydro alcoholic gel, and use of gloves), as a procedure of proven efficacy in the prophylaxis of the transmission of infectious diseases. Great importance was recently placed on the use of facial masks in hospitals, and of course, on hand washing [26].

A contaminated environment is one of the major risk factors for healthcare-associated infections, but some factors can definitely affect their incidence. It is widely documented that HCW’s hand decontamination practices, reduces the rates of hospital-acquired infections [27,28,29].

The Hospital Infection Control Committee drafted an internal evidence-based guideline for HCWs in 2002 and interventions were carried out in a Hand Washing Observation Program (HWOP) that started in 2015. Permanent monitoring of hand washing makes it possible to identify possible improvements in different health situations [30]. We collected data according to a standardized method used by the WHO, to observe the behavior of HCWs. More recently, some authors used video systems, but this use was not allowed in our hospital, because the recordings would inevitably involve the patient and harm privacy.

Adherence of HCWs, to recommended hand hygiene procedures, grew over the years, but is still insufficient, as compared to the lower limit suggested by the WHO (70%).

Despite these interventions and the adoption of a hospital procedure, we observed numerous differences among the operators of different departments.

The nurses are the healthcare personnel with the closest and most frequent contact with the patient. The rate of adherence of nurses is usually higher than the overall average percentage recorded in the wards, in any year under observation. It is likely that the nurses are educated and undergo adequate training for the described practice. In 2020, however, nurses worsened their performance due to understaffing, patient overflow, and reorganization in the wards.

The physicians who have more frequent contacts with patients are surgeons. The physicians hardly follow training courses in this area considering it not within their competence.

We observed that their own models influence the behavior of health professionals, confirming the benefit of leadership in HH improvement strategies [31].

In the general medical area, the procedure recorded the worst compliance, but it grew over time. The increase in adherence to the WHO protocol in the Medicine Unit probably might be due to parallel positive increase in favoring elements, as shown in Table 5. During the last two years, an improvement in the correct behavior in the departments of general medicine and a reduction in the departments of surgery was observed. In ICU, the differences in behavior observed over the years were due to the different emergencies or workload that occurred at different times. Intensive care personnel significantly changed their behavior by increasing the rate of adherence to the procedure during the pandemic period.

To explain these differences in behavior, we evaluated the critical elements regarding the organization of the department reported in the forms of the operator’s observation.

The presence among the nursing staff of a nurse as a member of the hospital’s infection control team was fundamental. Only deep awareness and training allow reaching the highest levels of adherence. For residents, adequate training on the subject is recommended. In fact, no specific subject is provided in the entire course of their studies on Medicine and Surgery.

To promote correct behavior, it can be proposed to entrust “adherence to the respect of hand hygiene” as a performance objective of all HCWs. It can also be suggested to do a practice run, because it is often found that HCW does not have a clear awareness of the 5 moments recommended by the WHO. They use the procedure more for defensive purposes than for preventive ones.

To achieve adequate levels of adherence, collaboration of the Hospital’s Pharmacy is essential for supplying the correct hydro alcoholic solutions and devices. The allocation of the hydro alcoholic gel dispenser in any room where patients were visited seemed to be more useful than the sink placed at the entrance of the hospital room.

A separate note should be written for discussing the use of gloves; this is being monitored since 2017. Healthcare personnel’s use gloves for defense against contagion rather than for prophylaxis towards the patient [32,33]. The use of gloves in pre-contact procedures was higher, especially for the healthcare assistant or the nursing assistant. Nevertheless, gloves do not change from one patient to the other, even though most hand hygiene guidelines recommend that gloves should be changed during each patient’s care [34]. The use of gloves, if not properly performed, could be harmful. It is reported that disinfection of gloved hands with pure alcohol, might substantially reduce the risk of transmission during multiple activities on the same patient [35].

The use of environmental decontamination procedures or disinfection of instruments might reduce the microbial load in the environment, but are never a substitute for hand hygiene, which remains the main vehicle of pathogens in hospitals [36,37,38,39].

Respect for correct behaviors in the hospital is not only a problem of technical knowledge of the procedures, but also personal education, psychological factors, and above all, organizational factors play an important role [40]. A high number of patients, a high workload or a high number of procedures to be quickly carried out might hinder compliance with the procedure. The patient/nurse ratio proved to be very important. Training courses and procedures can help to achieve better results when accompanied by multimodal interventions. Improvements can be reversed if changes are not permanent and do not continue [26,41,42,43].

New motivations in staff and patient involvement in hand hygiene should also be tested [44,45,46].

We can consider that the risk of contagion, through the hands of the healthcare staff, is effectively kept under control, since half of the observations concern nurses, and they carried out positive actions in about 70% of the cases observed.

Our work showed that the pandemic did not change the habits of health personnel regarding hand washing when approaching the patient. Despite the presence of guidelines, strong recommendations and the fear of COVID-19, unlike what could have been hypothesized, global adherence to correct hand hygiene procedures remained unchanged. In case of overcrowding of departments, it is not possible to guarantee correct hand washing. Health personnel practically reached maximum adherence to the procedure even before the pandemic. Only in ICUs, supported extensively by personnel and safety devices, a significant increase of the correct behavior was found, confirming that only major organizational changes could lead to an increase in the level of compliance.

Neither the percentage of washing with water nor the friction with gel changed. Health workers’ preference for hand washing rather than hydro alcoholic friction was also confirmed in 2020.

*Limitations and strengths of the study*. Observations were conducted in a single medium-sized hospital for a period of six consecutive years.

The constant monitoring over the years by the same observers reduced possible bias in the collection and analysis of data. These data would be used to evaluate the comparison with those that would be subsequently processed.

## 5. Conclusions

The comparison between the observations made in the years preceding the pandemic and the surveys carried out in 2020 did not show an overall increase in compliance with the procedure, despite fear of contagion.

Probably the overcrowding of the departments or under sizing of the staff did not make it possible to improve the performance of HCWs and therefore provide optimal care. Only in intensive care units, which are widely supported by personnel and safety devices, there was a significant increase in correct behavior.

Despite the health management involvement in the reduction of the transmissible charge by any means [41,47], every single operator should be ethically responsible for respecting the procedure.

Fear of contagion led to an increase in the use of gloves in pre-patient contact manoeuvres.

Despite what might be expected, adherence to the procedure did not increase. The medical staff probably already did the maximum.

More research on the psychological influence of COVID 19 on HCWs is needed.

## Figures and Tables

**Table 1:** Overall hospital hand hygiene compliance procedures per year.

Year	Opportunity	Hand Hygiene Actions	Compliance Rates (%)
2015	1158	HR 302	45
		HW 220	
		M 636	
2016	1450	HR 567	62
		HW 328	
		M 555	
2017	1771	HR 462	62
		HW 629	
		M 680	
2018	2351	HR 652	62
		HW 805	
		M 894	
2019	3595	HR 879	63
		HW 1380	
		M 1336	
2020	3169	HR 864	66
		HW 1216	
		M 1089	
	13,494		

Legend: OPPORTUNITY (situation that requires hand hygiene). HR = hand hygiene action by hand rubbing with an alcohol-based formula. HW = hand hygiene action by hand washing with soap and water. M = Missed—no hand hygiene action performed.

**Table 2:** Adherence to hand hygiene per professional category per year.

		Nurses			Attending Physician/Specialty Doctors			Residents/Students			Healthcare Assistant/Auxiliary Nurses		
Years	Opportunity	OPP	% Total OPP	Compliance (%)	OPP	% Total OPP	Compliance (%)	OPP	% Total opp	Compliance (%)	OPP	% Total OPP	Compliance (%)
2015	1158	509	44	57	196	17	47	340	29	31	113	10	29
2016	1450	641	44	65	286	20	65	272	19	64	251	17	52
2017	1771	879	50	67	182	10	65	309	17	51	401	23	57
2018	2351	1029	44	67	385	16	71	337	14	50	600	26	55
2019	3595	1700	47	69	692	19	62	340	10	38	863	24	60
2020	3169	1544	49	68	550	18	68	230	7	56	838	26	64
**Total**	**13** **,494**	**6302**			**2291**			**1828**			**3066**		

Legend: OPP= OPPORTUNITY (situation that requires hand hygiene). Compliance (%) = Actions/opportunities × 100.

**Table 3:** Adherence to hand hygiene by areas, per years.

		General Medicine Area		Surgical AREA		Intensive Care	
Years	Opportunity	Compliance (%)	OPP	Compliance (%)	OPP	Compliance (%)	OPP
2015	1158	56	240	40	699	58	219
2016	1450	48	454	68	722	67	274
2017	1771	47	431	71	885	58	455
2018	2351	57	1364	70	508	68	479
2019	3595	67	2035	57	1063	57	497
2020	3169	69	505	59	1556	73	1108

Legend: OPP= OPPORTUNITY (situation that requires hand hygiene). Compliance (%) = Actions/opportunities × 100.

**Table 4:** Adherence to hand hygiene per areas, per professional category, per years.

		General Medicine Area				Surgical Area				Intensive Care			
		Compliance (%)				Compliance (%)				Compliance (%)			
Years	Opportunity	NU	PHY	R	Others	NU	PHY	R	Others	NU	PHY	R	Others
2015	1158	39	63	74	25	57	45	41	16	73	53	34	40
2016	1450	51	52	32	50	70	69	76	52	74	73	60	55
2017	1771	57	29	31	49	72	90	78	62	71	47	28	48
2018	2351	61	61	39	51	70	81	82	67	80	69	45	53
2019	3595	74	65	40	66	62	60	48	48	69	56	22	60
2020	3169	71	55	64	74	58	71	62	53	77	71	42	77

Legend: NU = Nurse, PHY = Attending Physician/Specialty Doctors, R = Residents, Others = Healthcare assistant/auxiliary nurses.

**Table 5:** Favoring elements that can affect adherence to hand hygiene along years.

	2015			2016			2017			2018			2019			2020		
	M	S	I	M	S	I	M	S	I	M	S	I	M	S	I	M	S	I
Presence of hydro alcoholic gel dispenser on the wall in each room	0	0	1	0	0	1	1	0	1	1	1	1	1	1	1	1	1	1
Does the pharmacy promptly dispense the gel/soap in the required quantities?	0	0	1	0	0	1	0	0	1	0	1	1	1	1	1	1	1	1
Participation in training courses	1	1	1	1	1	1	1	1	1	1	0	1	1	0	0	0	0	0
With respect to the proportion of doctors/patients to be cared for (understaffing)	1	1	1	1	1	1	1	1	1	1	1	1	1	1	1	0	0	1
With respect to the proportion of nurses/patients to be assisted (understaffing)	0	0	0	0	0	0	0	0	1	0	0	0	0	0	1	0	0	1
With respect to the proportion of socio-health workers/patients to be cared for (understaffing)	0	0	0	0	0	0	0	0	0	0	0	0	0	0	0	0	0	1
Were the observations carried out constantly?	0	0	1	0	0	1	0	1	1	1	1	1	1	1	1	1	1	1
Did the Medical Directors and Head Nurses received the reports of the observations on hand washing carried out on their operators?	1	1	1	1	1	1	1	1	1	1	1	1	1	1	1	1	1	1
Active presence of at least 1 doctor/1 nurse from the ward in the hospital infection committee operating group	0	0	1	0	0	1	0	0	1	1	0	1	1	0	1	1	1	1
Active reporting of alert organisms or pathogenic germs	0	0	1	1	0	1	1	0	1	1	1	1	1	0	1	1	1	1
TOTAL	**3**	**3**	**8**	**4**	**3**	**8**	**5**	**4**	**9**	**7**	**6**	**8**	**8**	**5**	**8**	**6**	**6**	**9**

Legend: M = Medicine Area. S = Surgical Area. I = Intensive care Area.

## Data Availability

Data is available on request to the corresponding author (R.R.).

## References

[1] Melegari G., Iseppi R., Mariani M., Giuliani E., Caciagli V., Bertellini E., Messi P., Barbieri A. (2020). Keyboard Contamination in Intensive Care Unit: Is Cleaning Enough? Prospective Research of In Situ Effectiveness of a Tea Tree Oil (KTEO) Film. Adv. Exp. Med. Biol..

[2] Koscova J., Hurnikova Z., Pistl J. (2018). Degree of Bacterial Contamination of Mobile Phone and Computer Keyboard Surfaces and Efficacy of Disinfection with Chlorhexidine Digluconate and Triclosan to Its Reduction. Int. J. Environ. Res. Public Health.

[3] Otter J.A., Donskey C., Yezli S., Douthwaite S., Goldenberg S., Weber D.J. (2016). Transmission of SARS and MERS coronaviruses and influenza virus in healthcare settings: The possible role of dry surface contamination. J. Hosp. Infect..

[4] World Health Organization (WHO) (2009). Hand Hygiene Technical Reference Manual.

[5] Center for Disease Control and Prevention (CDC) Guidelines for Hand hygiene in Health-Care Setting. Hand Hygiene Guidance. https://www.cdc.gov/handhygiene/providers/guideline.html.

[6] Squeri R., Genovese C., Palamara M.A.R., Trimarchi G., La Fauci V. (2016). “Clean care is safer care”: Correct handwashing in the prevention of healthcare associated infections. Ann. Ig..

[7] Nobile C., Montuori P., Diaco E., Villari P. (2002). Healthcare personnel and hand decontamination in intensive care units: Knowledge, attitudes, and behaviour in Italy. J. Hosp. Infect..

[8] Bhagawati G. (2018). Get aware of hand hygiene: Implement it in your attitude. J. Educ. Health Promot..

[9] Salama O., Elweshahi H., El Raheem A.A. (2017). Knowledge, Attitudes and Compliance with Hand Hygiene Practices among Health Care Workers in Alexandria Main University Hospital. J. High Inst. Public Health.

[10] Randle J., Clarke M., Storr J. (2006). Hand hygiene compliance in healthcare workers. J. Hosp. Infect..

[11] Ataee R.A., Ataee M.H., Tavana A.M., Salesi M. (2017). Bacteriological Aspects of Hand Washing: A Key for Health Promotion and Infections Control. Int. J. Prev. Med..

[12] Chauhan B.F., Jeyaraman M.M., Mann A.S., Lys J., Skidmore B., Sibley K.M., Abou-Setta A.M., Zarychanksi R. (2017). Behavior change interventions and policies influencing primary healthcare professionals’ practice—An overview of reviews. Implement. Sci..

[13] Perry C., Chhatralia K., Damesick D., Hobden S., Volpe L. (2015). Behavioural Insights in Health Care. Nudging to Reduce Inefficiency and Waste.

[14] Dombrowski S.U., Campbell P., Frost H., Pollock A., McLellan J., MacGillivray S., Gavine A., Maxwell M., O’Carroll R., Cheyne H. (2016). Interventions for sustained healthcare professional behaviour change: A protocol for an overview of reviews. Syst. Rev..

[15] Johnson M.J., May C.R. (2015). Promoting professional behaviour change in healthcare: What interventions work, and why? A theory-led overview of systematic reviews. BMJ Open.

[16] Jamtvedt G., Young J.M., Kristoffersen D.T., O’Brien M.A.T., Oxman A.D. (2012). Audit and feedback: Effects on professional practice and health care outcomes. Cochrane Database Syst. Rev..

[17] Guo Y., Sims O., Qin W., Yang F. (2021). Factors Associated with Symptoms of Depression and Psychological Distress during the COVID-19 Pandemic. Behav. Sci..

[18] French M.T., Mortensen K., Timming A.R. (2020). Psychological Distress and Coronavirus Fears During the Initial Phase of the COVID-19 Pandemic in the United States. J. Ment. Health Policy Econ..

[19] Giorgi G., Lecca L.I., Alessio F., Finstad G.L., Bondanini G., Lulli L.G., Arcangeli G., Mucci N. (2020). COVID-19-Related Mental Health Effects in the Workplace: A Narrative Review. Int. J. Environ. Res. Public Health.

[20] Nkire N., Nwachukwu I., Shalaby R., Hrabok M., Vuong W., Gusnowski A., Surood S., Greenshaw A.J., Agyapong V.I.O. (2021). COVID-19 pandemic: Influence of relationship status on stress, anxiety, and depression in Canada. Ir. J. Psychol. Med..

[21] Nwachukwu I., Nkire N., Shalaby R., Hrabok M., Vuong W., Gusnowski A., Surood S., Urichuk L., Greenshaw A.J., Agyapong V.I. (2020). COVID-19 Pandemic: Age-Related Differences in Measures of Stress, Anxiety and Depression in Canada. Int. J. Environ. Res. Public Health.

[22] El-Hage W., Hingray C., Lemogne C., Yrondi A., Brunault P., Bienvenu T., Etain B., Paquet C., Gohier B., Bennabi D. (2020). Les professionnels de santé face à la pandémie de la maladie à coronavirus (COVID-19): Quels risques pour leur santé mentale?. L’Encéphale.

[23] Preti E., Di Mattei V., Perego G., Ferrari F., Mazzetti M., Taranto P., Di Pierro R., Madeddu F., Calati R. (2020). The Psychological Impact of Epidemic and Pandemic Outbreaks on Healthcare Workers: Rapid Review of the Evidence. Curr. Psychiatry Rep..

[24] Carmassi C., Foghi C., Dell’Oste V., Cordone A., Bertelloni C.A., Bui E., Dell’Osso L. (2020). PTSD symptoms in healthcare workers facing the three coronavirus outbreaks: What can we expect after the COVID-19 pandemic. Psychiatry Res..

[25] Pollock A., Campbell P., Cheyne J., Cowie J., Davis B., McCallum J., McGill K., Elders A., Hagen S., McClurg D. (2020). Interventions to support the resilience and mental health of frontline health and social care professionals during and after a disease outbreak, epidemic or pandemic: A mixed methods systematic review. Cochrane Database Syst. Rev..

[26] Alzyood M., Jackson D., Aveyard H., Brooke J. (2020). COVID-19 reinforces the importance of handwashing. J. Clin. Nurs..

[27] Sickbert-Bennett E.E., DiBiase L.M., Willis T.M.S., Wolak E.S., Weber D.J., Rutala W.A. (2016). Reduction of Healthcare-Associated Infections by Exceeding High Compliance with Hand Hygiene Practices. Emerg. Infect. Dis..

[28] Ragusa R., Corsaro L.S., Frazzetto E., Bertino E., Bellia M.A., Bertino G. (2020). Hepatitis C Virus Infection in Children and Pregnant Women: An Updated Review of the Literature on Screening and Treatments. Am. J. Perinatol. Rep..

[29] Koff M.D., Brown J.R., Marshall E.J., O’Malley A.J., Jensen J.T., Heard S.O., Longtine K., O’Neill M., Longtine J., Houston D. (2016). Frequency of Hand Decontamination of Intraoperative Providers and Reduction of Postoperative Healthcare-Associated Infections: A Randomized Clinical Trial of a Novel Hand Hygiene System. Infect. Control Hosp. Epidemiol..

[30] Ragusa R., Giorgianni G., Sciacca A., Rametta S., La Verde M., Mulè S. (2018). Healthcare-associated Clostridium dif-ficile infection: From the study of the observed cases to effective HA-CDI prevention measures. An observational study in a teaching hospital in Sicily. J. Prev. Med. Hyg..

[31] Huis A., Holleman G., Van Achterberg T., Grol R., Schoonhoven L., Hulscher M. (2013). Explaining the effects of two different strategies for promoting hand hygiene in hospital nurses: A process evaluation alongside a cluster randomised controlled trial. Implement. Sci..

[32] Marranzano M., Ragusa R., Platania M., Faro G., Coniglio M. (2013). Knowledge, attitudes and practices towards patients with HIV/AIDS in staff nurses in one university hospital in Sicily. Epidemiol. Biostat. Public Health.

[33] Rapisarda V., Loreto C., Vitale E., Matera S., Ragusa R., Coco G., Rapisarda L., Ledda C. (2019). Incidence of sharp and needle-stick injuries and mucocutaneous blood exposure among healthcare workers. Future Microbiol..

[34] Trick W.E., Vernon M.O., Welbel S.F., De Marais P., Hayden M.K., Weinstein R.A. (2007). Chicago Antimicrobial Resistance Project. Multicenter Intervention Program to Increase Adherence to Hand Hygiene Recommendations and Glove Use and to Reduce the Incidence of Antimicrobial Resistance. Infect. Control Hosp. Epidemiol..

[35] Kampf G., Lemmen S. (2017). Disinfection of gloved hands for multiple activities with indicated glove use on the same patient. J. Hosp. Infect..

[36] Ragusa R., Lombardo A., Bruno A., Sciacca A., Lupo L. (2016). Environmental Biodecontamination: When a Procedure Performed by the Nursing Staff has an Economic Impact in ICU Rooms. J. Nurs. Care.

[37] Castaldi S., Ragusa R. (2017). HTA applicato all’Igiene ospedaliera. J. Prev. Med. Hyg..

[38] Ragusa R., Giorgianni G., Lombardo A., Faro G., Lupo L., Marranzano M. (2020). Automated UV- C LED stethoscope decontamination: A useful barrier in the time of COVID-19. East. J. Med..

[39] Ragusa R., Giorgianni G., Faro G., Lazzara A., Bellia M.A., Marranzano M. (2019). Are Visitors Dangerous Carriers of Pathogens in The Hospital? An Observational Study in an University Hospital in Sicily. Hosp. Top..

[40] Von Lengerke T., Ebadi E., Schock B., Krauth C., Lange K., Stahmeyer J.T., Chaberny I.F. (2019). Impact of psychologically tailored hand hygiene interventions on nosocomial infections with multidrug-resistant organisms: Results of the cluster-randomized controlled trial PSYGIENE. Antimicrob. Resist. Infect. Control.

[41] Baccolini V., D’Egidio V., De Soccio P., Migliara G., Massimi A., Alessandri F., Tellan G., Marzuillo C., De Vito C., Ranieri M.V. (2019). Effectiveness over time of a multimodal intervention to improve compliance with standard hygiene precautions in an intensive care unit of a large teaching hospital. Antimicrob. Resist. Infect. Control.

[42] Hinkin J. (2002). Hand decontamination: What interventions improve compliance?. Edtna-Erca J..

[43] Landers T., Abusalem S., Coty M.-B., Bingham J. (2012). Patient-centered hand hygiene: The next step in infection prevention. Am. J. Infect. Control.

[44] Lutze B., Chaberny I.F., Graf K., Krauth C., Lange K., Schwadtke L., Stahmeyer J., Von Lengerke T. (2017). Intensive care physicians’ and nurses’ perception that hand hygiene prevents pathogen transmission: Belief strength and associations with other cognitive factors. J. Health Psychol..

[45] White K.M., Jimmieson N.L., Obst P.L., Graves N., Barnett A., Cockshaw W., Gee P., Haneman L., Page K., Campbell M. (2015). Using a theory of planned behaviour framework to explore hand hygiene beliefs at the ‘5 critical moments’ among Australian hospital-based nurses. BMC Health Serv. Res..

[46] Rahim M.H.A., Ibrahim M.I., Noor S.S.M., Fadzil N.M. (2021). Predictors of Self-Reported Hand Hygiene Performance among Nurses at Tertiary Care Hospitals in East Coast Malaysia. Int. J. Environ. Res. Public Health.

[47] Pittet D., Hugonnet S., Harbarth S., Mourouga P., Sauvan V., Touveneau S., Perneger T.V. (2000). Effectiveness of a hospital-wide programme to improve compliance with hand hygiene. Lancet.

